# Polystyrene Microplastics Exposure: An Insight into Multiple Organ Histological Alterations, Oxidative Stress and Neurotoxicity in Javanese Medaka Fish (*Oryzias javanicus* Bleeker, 1854)

**DOI:** 10.3390/ijerph18189449

**Published:** 2021-09-07

**Authors:** Sunusi Usman, Ahmad Faizal Abdull Razis, Khozirah Shaari, Mohammad Noor Azmai Amal, Mohd Zamri Saad, Nurulfiza Mat Isa, Muhammad Farhan Nazarudin

**Affiliations:** 1 Natural Medicines and Products Research Laboratory, Institute of Bioscience, Universiti Putra Malaysia, Serdang 43400 UPM, Selangor, Malaysia; usunusi.bch@buk.edu.ng (S.U.); khozirah@upm.edu.my (K.S.); 2 Department of Food Science, Faculty of Food Science and Technology, Universiti Putra Malaysia, Serdang 43400 UPM, Selangor, Malaysia; 3 Department of Chemistry, Faculty of Science, Universiti Putra Malaysia, Serdang 43400 UPM, Selangor, Malaysia; 4 Department of Biology, Faculty of Science, Universiti Putra Malaysia, Serdang 43400 UPM, Selangor, Malaysia; mnamal@upm.edu.my; 5 Aquatic Animal Health and Therapeutics Laboratory (Aqua Health), Institute of Bioscience, Universiti Putra Malaysia, Serdang 43400 UPM, Selangor, Malaysia; mzamri@upm.edu.my (M.Z.S.); m_farhannaza@upm.edu.my (M.F.N.); 6 Department of Veterinary Laboratory Diagnosis, Faculty of Veterinary Medicine, Universiti Putra Malaysia, Serdang 43400 UPM, Selangor, Malaysia; 7 Department of Cell and Molecular Biology, Faculty of Biotechnology and Biomolecular Sciences, Universiti Putra Malaysia, Serdang 43400 UPM, Selangor, Malaysia; nurulfiza@upm.edu.my; 8 Laboratory of Vaccines and Biomolecules (VacBio), Institute of Bioscience, Universiti Putra Malaysia, Serdang 43400 UPM, Selangor, Malaysia

**Keywords:** polystyrene microplastics, histological alterations, oxidative stress, neurotoxicity, Javanese medaka fish

## Abstract

Microplastics (MPs) have become pollutants of concern due to their unknown human health effect and negative impact on terrestrial and aquatic ecosystems. There is increasing number of experimental research on MPs globally with its effects not fully understood; recent animal studies explore its effects on the intestines, yet on other vital organs. Javanese medaka fish was exposed to polystyrene microplastics (PS-MPs) beads for a period of 21 days. Histological alterations, intestinal oxidative stress, permeability and neurotoxicity were evaluated. Significant inflammatory changes and tissue damage were observed in the intestine, liver and kidney. Intestinal oxidative stress and permeability were found to be significantly increased. In the brain, neurotoxicity characterised by a significant induction of oxidative stress, lipid peroxidation and the inhibition of acetylcholinesterase enzyme were elucidated. This study provided an insight into the multiple organ effect of microplastics exposure, necessitating further exploration and identification of biomarkers to be utilised for biomonitoring population at risk in the future.

## 1. Introduction

Plastics versatility, light weight and the low cost of production have made them invaluable in our society, thus fuelling their global demand [1]. Plastic production has dramatically risen worldwide [2]. As of 2019, stood at 368 million metric tons (MT), with the figure expected to double in the next 20 years. Coastal countries generate about 275 million MT of plastics out of which 4.8 to 12.7 enter the ocean [3]. MPs synthesised primarily in micro-sized form (5 µm or less), and those emanating from the degradation of larger plastics are mostly discharged into the aquatic environment due to indiscriminate disposal and accidental spills [4]. MPs were found in all the seas [5] ingested by marine organisms of all sizes. Their small size and non-biodegradable nature are highly important [6], providing them with the ability to become transferable and accessible to a wide range of marine organisms across the food web [7], thus becoming a danger to environmental health, ecological safety and human health [8,9].

Microplastics were found to bioaccumulate following ingestion by aquatic organisms including fish and marine mammals, this enable their subsequent transfer along the trophic level [10,11]. MPs possess the inherent capability to be transferred from digestive tract and/or gills into the circulatory system [12] with the translocation mechanism not fully understood. MPs have been observed in organs and tissues of crustaceans and fishes [13]. Fluorescent PS-MPs of 5 µm and 20 µm have been demonstrated to accumulate in the liver, kidney and gut of mice [14].

Humans are exposed to MPs, especially with the increasing consumption of sea food as a major dietary protein. About 39,000 to 52,000 particles per person per year of MPs are estimated to be consumed through food [15]. Additionally, MPs were found in drinking water, which necessitated the World Health Organisation to call for an urgent exploration into microplastics pollution and its consequent effect on human health [16].

Although recognised as an environmental health hazard due to their effects on the environment and the biota, research on MPs is generally limited, as less than a quarter of the 192 countries in the world has so far conducted research in that respect [17]. For instance, till date there is a paucity of knowledge on the adverse effects of consuming MPs containing marine organisms [18]. This has spurred an array of animal studies in an attempt to explore the possible effects and mechanisms to which microplastics affect living organisms. Most studies conducted observed the effects of MPs on growth and reproduction [19,20,21]. Of the few studies on the gut, the effect revealed inflammatory changes and the induction of oxidative stress [21,22,23,24,25].

In an attempt to investigate the effects of oxidative stress using markers of lipid peroxidation (MDA, lipofuscin and neutral lipid), no evidence of oxidant damage was found [26,27], thus supporting the literature limiting the effect of MPs on the induction of oxidative stress alone [26,28,29,30]. However, MPs ingestion and accumulation in the gut have been shown to impair intestinal barrier function [29]. Additionally, oxidative stress being a condition predominated by the formation of highly reactive oxygen species that can interact with cellular structures causing damage [31] may not be only limited to the gut. The brain particularly is said to be at risk of lipid peroxidation and subsequent pathological processes in situations of impaired antioxidant defence mechanisms due to its high oxygen demand [32,33]. This is evident in neurodegenerative diseases, including Parkinson’s disease (PD), Huntington’s Disease (HD), Alzheimer’s disease (AD) and amyotrophic lateral sclerosis (ALS), which presents with evidence of lipid peroxidation implicated by oxidative stress and damage [34].

The aim of this study was to evaluate the impact of PS-MPs exposure on Javanese medaka fish (*Oryzias javanicus* Bleeker, 1854). Several organisms were used in ecotoxicological studies as biomonitoring agents. Javanese medaka (*O. javanicus*), as representative of the mangrove ecosystem is capable of adapting to fresh and brackish/sea water [35], is a close relative of the well-established Japanese medaka, a fish native to the estuaries of Malaysian peninsula, Thailand, Indonesia and Singapore. It is considered a promising marine model test fish [36,37] and a potential test organism [38].

Histological alterations following polystyrene microplastics (PS-MPs) exposure in various organs (intestine, liver, kidney and brain) were investigated. Additionally, intestinal oxidative stress and permeability were assessed using antioxidant enzymes (Catalase and Superoxide dismutase) and D-lactate, respectively. Additionally, brain oxidative stress, oxidative damage and neurotoxicity were assessed using catalase, superoxide dismutase, malondialdehyde, acetylcholine and acetylcholinesterase, respectively.

## 2. Materials and Methods

### 2.1. MPs Used in the Study

#### Form

Polystyrene microplastics beads (5 µm SD < 0.1 µm coefficient variance < 2%; aqueous suspension; white colour; 0% cross-linked; 10% solids; 1.0 g/cm^3^ density) purchased from Sigma Aldrich (Industriestrasse, Buchs (SG), Switzerland) were used. Polystyrene was chosen based on previous literatures using different animal models [14,29], with the 5 µm size considered as it can be ingested by all aquatic organisms due to its small size [6], and will be easily accessible to Javanese medaka fish to allow for exploration of its exposure effect. Additionally, MPs fibres, debris or particles within the range 1 µm–5 mm have entered the body of humans with risk still unknown [39].

Size and morphology were checked using scanning electron microscopy (SEM), (JOEL JSM 6400 SEM Japan) and the chemical composition was confirmed using Fourier transform infrared spectroscopy (FTIR) (Thermo Fisher Scientific (Waltham, MA, USA), Nicolet 6700). PS-MPs suspension was prepared using UV sterilised dechlorinated tap water and sonicated prior to use.

### 2.2. Medaka Fish Maintenance and PS-MPs Exposure

Wild strain healthy adult Javanese medaka fish (0.5–0.7 g in weight; 3–4 cm in length; both sexes) were collected from the Sepang estuary (2°37′15.38″ N, 101°42′38.33″ E) Malaysia, and maintained in a semi static circulating system under suitable condition (temperature; 26 ± 1 °C, pH; 7.8–8.0, DO; 5.5–6.0 mg L^−1^, photoperiod; 14:10 h light/dark cycle) measured and recorded daily [40]. Fish were acclimatised in 15 L glass tanks for 2 weeks at Aquatic Animal Health and Therapeutics Laboratory (Aqua Health), Institute of Bioscience, Universiti Putra Malaysia. During the same period, the salinity was gradually reduced daily by 2 ppt from the Sepang estuary value of 14.5 ± 0.5 ppt for its adaptability with the water used in the experiment, this is in accordance with previous research [41], and due to the adaptability of Javanese medaka to live a normal life and reproduce in both fresh water and brackish/Sea water [35,42]. Fish were fed with commercial feed (Aquadene) at 1.0% body weight twice daily. The acclimatisation was in UV-sterilised well aerated dechlorinated tap water refreshed every 48 h.

Acclimatised Javanese medaka fish were randomly assigned into control group and three PS-MPs treatment groups and exposed for a period of 21 days (5 fish and 1 L test solution in each glass tank). In the treatment groups, fish were exposed to test solution prepared by dispersing PS-MPs in UV-sterilised well aerated dechlorinated tap water to achieve a final PS-MPs concentration of 100 µg/L (1.46 × 10^3^, MP-LOW), 500 µg/L (7.3 × 10^3^, MP-MED) and 1000 µg/L (1.46 × 10^4^, MP-HIGH). The exposure concentrations of the treatments were chosen based on previous literature [23,29]. Control group fish were exposed to PS-MPs free UV-sterilised dechlorinated tap water. Test solutions in each tank of the treatment groups and dechlorinated tap water in the control group were refreshed every 48 h. Tanks were placed under continuous aeration to maintain dissolved oxygen within optimal range and ensure the even dispersion of PS-MPs particles. Other conditions were maintained as during the acclimatisation period. The research was performed with the approval and in accordance with the guidelines of the Institutional Animal Care and Use Committee of Universiti Putra Malaysia (UPM/IACUC/AUP-R021/2020).

Medaka fish were exposed for a duration of 21 days. Subsequently, they were sampled in the same day and washed three times with purified water (MilliQ), followed by euthanization through immersion in ice water at 4 °C. The intestine, liver, kidney and brain were dissected and treated differently for histological and biochemical analyses.

### 2.3. Histological Analysis

There were 20 fishes used for histological analyses. This is represented by 5 replicates in each of the control and the three PS-MPs treatment groups, with each replicate having 1 Javanese medaka. Samples including gut, liver, kidney and brain dissected and fixed in 10% neutral phosphate buffered formalin. Standard techniques were followed to process the tissue samples for histological analysis after their fixation. In brief, the intestines, liver, kidney and brain tissues were cut at 5 μm with a microtome. They were then embedded in paraffin for 2 min in xylene. Subsequently, the samples were hydrated by the serial transfer of the slides through 100% ethanol, 95% and finally 70% ethanol for 2 min each. This was followed by rinsing the slides with running water for a minimum of 2 min. Haematoxylin solution was used to stain the samples for 3 min, and were then washed again with water and subsequently stained with eosin for 2 min. The samples were then dehydrated by immersion 20 times in 95% ethanol and subsequently in 95% and 100% ethanol for 2 min each. Xylene was used to clean the samples for 2 min by immersion which is followed by adding a drop of premount solution over the slide with the subsequent addition of a coverslip. Each replicate has 10 slices. Five different fields were selected at random from each slice (200×) under a Leica™ (Leica Microsystem, Germany) to detect and photograph the presence of histological lesions, of which a minimum of three fields were required to have lesions and to be considered as histologically altered. Observer remained blinded for treatment and control groups during histological observations [29,43]. The observed lesions were presented as a percentage of tissue slices with obvious lesion out of 10 slices in each replicate, with mean percentage and standard deviation of each group computed.

### 2.4. The Determination of Intestinal Oxidative Stress and Permeability

Catalase (CAT) and total superoxide dismutase (T-SOD) commercial kits purchased from Elabscience (Houston, TX, USA) were used to measure oxidative stress; D-lactate (Abcam, Cambridge, UK) was used to determine intestinal permeability. For each of the control and the three PS-MPs treatment groups, there were four biological replicates, with each replicate comprised of 15 fish (total of 240 medaka fish). Pooled intestines of the 15 fishes from each replicate were homogenised for detection, protein content quantification was determined by BCA method using BCA Protein Assay Kit (Thermo Fisher Scientific (Waltham, MA, USA)), and the experimental procedures conducted were in accordance with the manufacturers’ protocol.

### 2.5. The Determination of Brain Oxidative Stress and Neurotoxicity

Brain tissues of fish from Section 2.4 above were at the same time dissected and pooled for detection. Malondialdehyde (MDA) (Fine Test, Wuhan, HB, China), CAT, T-SOD (Elabscience, Houston, TX, USA), acetylcholine (ACh) (Elabscience, Houston, TX, USA) and acetylcholinesterase (AChE) (Cusbio, Houston, TX, USA) were used to measure brain antioxidant activity, oxidant damage and neurotoxicity. Experimental operations were conducted following manufacturers’ protocol.

### 2.6. Statistical Analysis

Data generated were analysed using the IBM SPSS Statistics 27.0 computer program (SPSS, Chicago, IL, USA) and presented as mean ± standard deviation. One-way analysis of variance (ANOVA) was used to determine the difference between means, while the levels of significance were declared at *p* < 0.05 using Tukey’s honestly significance difference (HSD) post-hoc test. Prior to conducting one-way ANOVA, the normality and homogeneity of variance of the data were computed and confirmed to be appropriate for the test by normal plot of probability, versus fit, histogram and versus order.

## 3. Results

### 3.1. The Physical and Chemical Confirmation of PS-MPs Used in the Study

**Figure 1:**
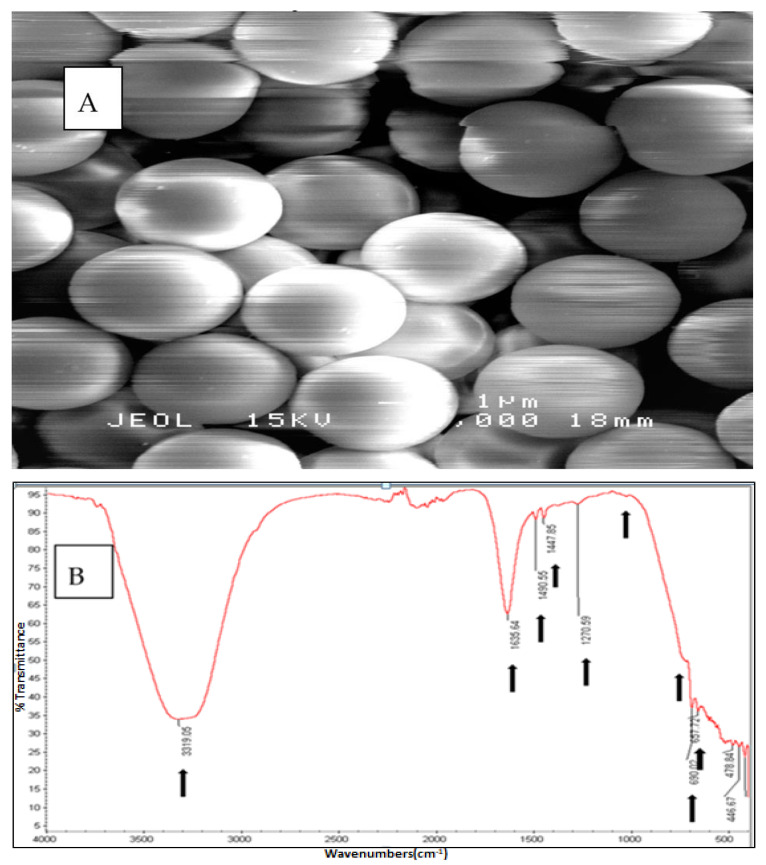
(**A**) SEM image of PS-MPs (0.5 µm) and (**B**) FTIR spectrum of polystyrene microplastics beads used in this study.

The PS-MPs beads were confirmed as spherical in shape and of homogenous size and distribution (Figure 1A). Chemical composition was confirmed using FTIR, the absorbance peaks fall within a wide range of spectral scale. The spectral peaks qualitatively confirmed the microbeads as polystyrene based on the vibration positions indicated with arrows (Figure 1B).

### 3.2. Histological Alterations in the Intestines, Liver, Kidney and Brain

**Figure 2:**
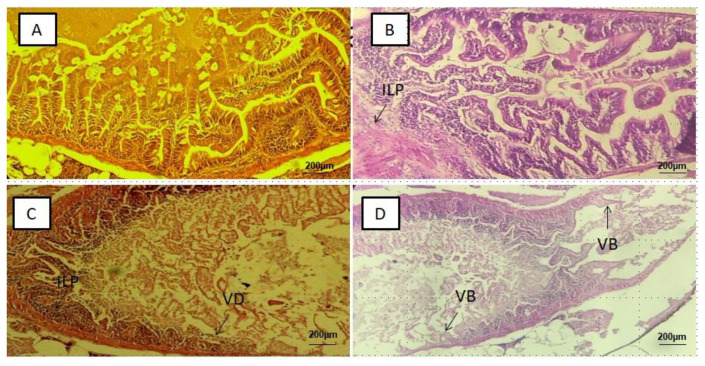
Representative photomicrograph of intestine: (**A**) Control group with no abnormality, (**B**) MP-LOW group showing inflammation of lamina propria (ILP), (**C**) MP-MED group showing inflammation of lamina propria (ILP), and villi destruction (VD) and (**D**) MP-HIGH group showing villous blunting (VB).

**Figure 3:**
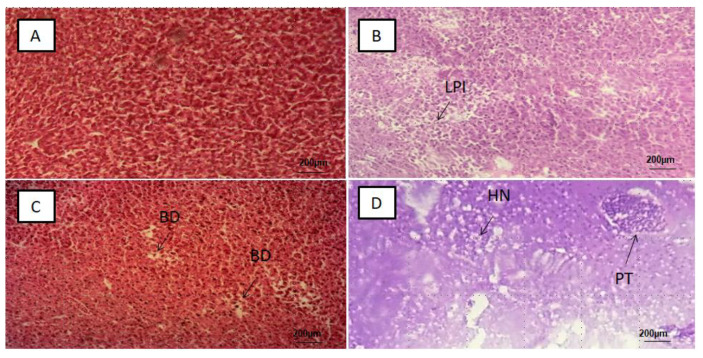
Representative photomicrograph of liver tissue: (**A**) Control group with normal liver tissue, (**B**) MP-LOW group showing liver parenchymal inflammation (LPI), (**C**) MP-MED group showing ballooning degeneration of hepatocytes (BD), and (**D**) MP-HIGH group showing hepatic necrosis (HN) and portal triaditis (TN).

**Figure 4:**
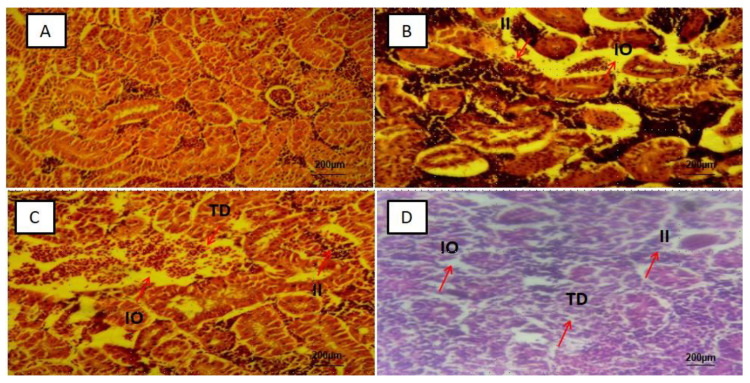
Representative photomicrograph of kidney tissue: (**A**) control group with normal tissue, (**B**) MP-LOW, (**C**) MP-MED, and (**D**) MP-HIGH, showing interstitial inflammation (II), interstitial oedema (IO) and tubular destruction (TD).

**Figure 5:**
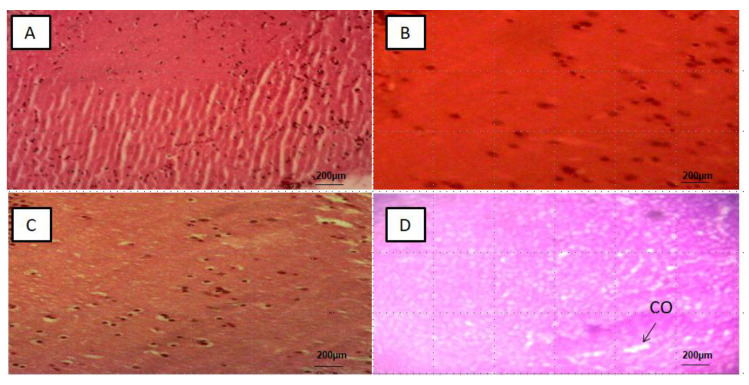
Representative photomicrograph of brain tissue: (**A**) Control group with normal tissue, (**B**) MP-LOW, no lesion, (**C**) MP-MED, no lesion and (**D**) MP-HIGH group showing cerebral oedema (CO).

**Figure 6:**
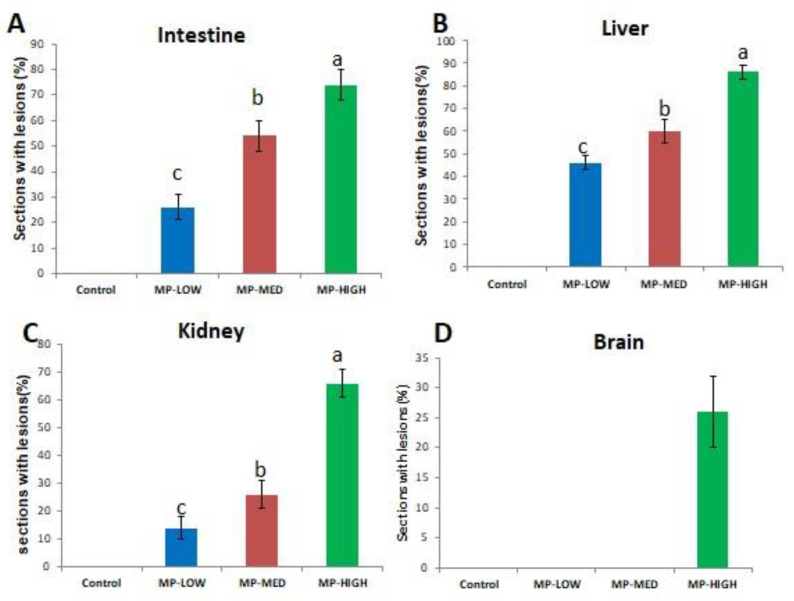
Percentages of histological sections with alteration(s); (**A**) intestine, (**B**) liver, (**C**) kidney and (**D**) brain of Javanese medaka fish in MP-LOW, MP-MED and MP-HIGH groups. PS-MPs exposed groups with different alphabets (a, b and c) denotes significant difference (*p*-value < 0.05). Data presented in percentage mean ± SD of 5 biological replicates (10 slices in each replicate).

Alteration was not detected in the intestine, liver, kidney and brain tissue slices of the control groups (Figure 2A, Figure 3A, Figure 4A and Figure 5A), respectively. In the PS-MPs exposed groups, the intestines showed villi blunting and destruction, inflammation and lymphocytic infiltration of the lamina propria (Figure 2B–D). Of the total number of 50 slices (10 slices per replicate, and 5 replicates in each group) the mean percentage and standard deviation of intestinal tissue slices with alteration observed among the three exposed groups was found to be significant (*p*-value < 0.05), and recorded as MP-HIGH (74 ± 6%), MP-MED (54 ± 6%) and MP-LOW (26 ± 5%) (Figure 6A).

The liver tissue slices showed lobar inflammation, portal triaditis, ballooning degeneration and hepatic necrosis (Figure 3B–D). Significant difference (*p*-value < 0.05) was found between the PS-MPs exposed groups, the mean percentages and standard deviations of the 50 slices in each of the PS-MPs exposed group with alterations was found to be MP-HIGH (86 ± 3%), MP-MED (60 ± 5%) and MP-LOW (46 ± 3%) (Figure 6B).

The kidney slices observed showed interstitial inflammation, interstitial oedema and tubular destruction (Figure 4B–D), significant difference (*p*-value < 0.05) in mean percentages of tissue alterations of the 50 slices observed in each PS-MPs exposed group was found and represented as MP-HIGH (66 ± 4%), MP-MED (26 ± 5%) and MP-LOW (14 ± 4%) (Figure 6C). The MP-LOW and MP-MED brain tissue slices showed no obvious abnormality (Figure 5B,C). However, 26 ± 6% of the 50 slices of MP-HIGH sections showed features of cerebral oedema (Figure 6D).

### 3.3. Intestinal Oxidative Stress and Increased Permeability Induced by PS-MPs

**Figure 7:**
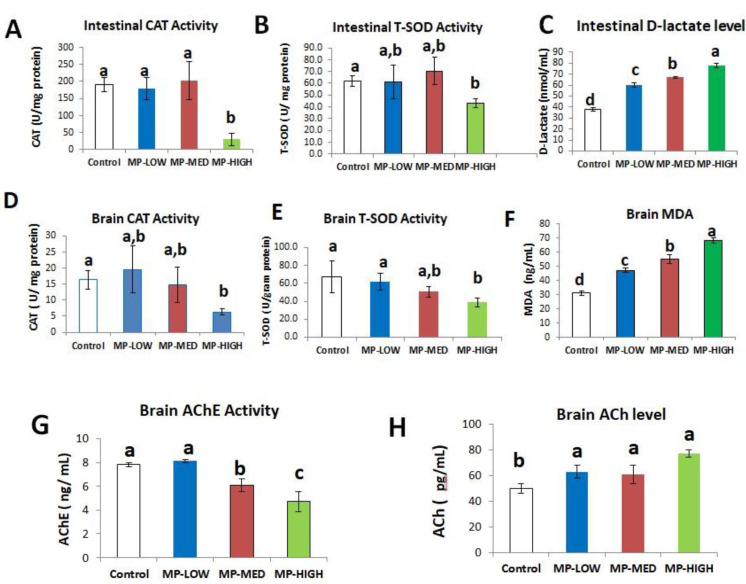
Bar charts of biomarkers of intestinal oxidative stress (**A**,**B**), intestinal permeability (**C**), brain oxidative stress (**D**,**E**), lipid peroxidation (**F**) and neurotoxicity (**G**,**H**). Groups with different alphabets denotes significant difference (*p*-value < 0.05). Data presented in mean ± SD of 4 biological replicates.

Intestinal oxidative stress was significantly increased in the PS-MPs exposed groups. CAT activity increased in MP-MED group (202 ± 56 U/mg protein) and decreased in the MP-HIGH group. Significant difference (*p*-value < 0.05) was found between the three PS-MPs exposed groups and the control (191 ± 22 U/mg protein) (Figure 7A). Similarly, T-SOD activity slightly increased in the MP-MED group (70.5 ± 12 U/mg protein) and decreased in the MP-HIGH group (43 ± 4 U/mg protein), significant difference (*p*-value < 0.05) was found between MP-HIGH and the control (61.8 ± 5 U/mg protein) (Figure 7B).

Intestinal permeability was significantly increased. Measured using D-lactate, significant difference (*p*-value < 0.05) was found between the three treatments groups [(MP-LOW (60 ± 2 nmol/mL), MP-MED (67 ± 1 nmol/mL) and MP-HIGH (78 ± 2 nmol/mL)] and also between the treatments and the control (38 ± 2 nmol/mL) (Figure 7C).

### 3.4. Brain Oxidative Stress and Neurotoxicity

Increase in brain oxidative stress and oxidative damage was found in PS-MPs exposed groups assessed by the activity and level of CAT, T-SOD and MDA, respectively. CAT activity in U/mg protein was found to increase slightly in the MP-LOW group (20 ± 7) and decrease in the MP-MED group (15 ± 6). Significant decrease (*p*-value < 0.05) was found between MP-HIGH (6 ± 1) and the control (16 ± 1) (Figure 7D). T-SOD activity showed a progressive decline in MP-LOW (62 ± 10 U/g protein), MP-MED (50.8 ± 6 U/g protein) and MP-HIGH (38.8 ± 5 U/g protein) groups, with significant difference (*p*-value < 0.05) found between the MP-HIGH compared to the MP-LOW and the control (67 ± 18 U/g protein) (Figure 7E). The MDA level in the brain showed a significant progressive increase in the MP-LOW (47 ± 2 ng/mL), MP-MED (55 ± 3 ng/mL) and MP-HIGH (68 ± 2 ng/mL) groups, with significant difference (*p*-value < 0.05) found between the three treatments and between the treatments and the control (Figure 7F).

Neurotoxicity was detected in the PS-MPs exposed groups. A significant decrease in the activity of AChE (*p*-value < 0.05) was found between the MP-MED (6 ± 1 µg/mL) and MP-HIGH (5 ± 1 µg/mL) groups and between the same groups and the control (8 µg/mL) (Figure 7G). Conversely, the level of ACh progressively increases in the exposed groups. A statistically significant increase (*p*-value < 0.05) was found between the three treatments and the control (Figure 7H).

## 4. Discussion

The inflammatory changes, villi blunting and damage observed in the intestines of Javanese medaka fish (Figure 2B–D) are in agreement with the findings of studies that explored the effects of microplastics on European sea bass, gold fish, mice and zebra fish [11,23,24,29,44]. Lesions are more frequent in the highest exposure concentration group similar to the findings of Qiao et al. [24]. However, contrary to our finding, MPs were not found to have effect on *S. aurata* following dietary exposure [45].

Lesions in the liver are more frequent in the highest PS-MPs exposed group, with inflammation, ballooning degeneration, hepatocytes necrosis and portal triaditis as the major observed alterations (Figure 3B–D). Similarly, histological changes of hyperaemia, dilated sinusoids and hydrophobic vacuolisation were found in the liver sections of Tilapia fish. However, unlike in our study, the damage was attributed to MPs rough shape [24].

In the kidneys, the interstitial inflammation, tubular oedema and destruction was observed (Figure 4B–D), this may be attributed to the capability of MPs to transverse the digestive system into the circulatory system [12] and accumulates in the organs [10,11] and also due to recent finding exhibited by kidney cells treated with PS-MPs which showed higher level of mitochondrial ROS, ER stress-related proteins and inflammation related protein [46].

The histological lesion of cerebral oedema was observed in the brain sections of Javanese medaka fish exposed to the highest concentration of PS-MPs. This may be due to increase in oxidative stress and oxidant damage observed in this study, which has been found to be implicated in several pathological conditions of the nervous system [32,33,34]. Another possible reason may be due to the ability of micro and nano-plastics taken up by aquatic organisms and mammals to reach the brain and exert a range of neurotoxic effects [47], however there is need for further exploration on the interaction of MPs and nervous system particularly the brain, as most studies focused on the gastrointestinal system.

Intestinal oxidative stress (Figure 7A,B) and permeability (Figure 7C) were significantly increased. This is in agreement to the study conducted on fish of different species [19,29,30]. Although studies have related impaired intestinal barrier function and permeability with inflammation and oxidative stress in the gut [48], inflammatory diseases within and outside the gastrointestinal system, such as irritable bowel disease, irritable bowel syndrome, alcoholic liver disease, liver cirrhosis and chronic kidney disease were implicated to impair intestinal barrier function [49]. Furthermore, diet, host immunity and microbiota were among the important regulators of the mucosal barrier function, and are essential for the regulation of intestinal permeability and the overall maintenance of normal gastrointestinal functions [50]. The increased intestinal permeability observed in our study may be as a result of multiple organ affectation following PS-MPs exposure. In addition to gut inflammation, oxidative stress, kidney and liver alterations; brain inflammation and neurotoxicity may also be a contributory factor, as the gut brain axis was found to be an important pathway of interaction and physiological control between the brain and the intestine. A notable example is in oxidative stress laden neurodegenerative diseases found to alter gut function and microbiota, which in turn can reversely increase brain inflammation and reactive oxygen species [51].

The significant oxidative stress and oxidant damage observed in this study (Figure 7D–F) are in agreement with the findings of the significant inhibition of SOD and CAT in *Clarias* *gariepinus* exposed to polyvinyl chloride, depicting the dramatic generation of reactive oxygen species leading to low antioxidant enzyme activity and significant increase in the MDA level [52].

Neurotoxicity was found as a consequent effect of PS-MPs exposure (Figure 7G,H). This is similar to the findings in *Oreochromis niloticus* exposed to PS-MPs, with observed suppression in the catalytic activity of AChE in the brain. However, no evidence of lipid peroxidation was found unlike in our study [53]. The observed inhibition of AChE in this study can be attributed to the induction of oxidative stress and corresponding oxidant damage in the brain. AChE as a key enzyme of the nervous system was recognised as a human biomarker of pesticides poisoning due to its sensitivity, ease of measurement and dose dependent behaviour to pollutant exposure [54].

## 5. Conclusions

This study provides an insight into PS-MPs exposure causing histological and biochemical alterations in the gastrointestinal system and other vital organs. The underlying mechanisms of how MPs affect multiple organs and the dose and duration of exposure that may lead to the development of diseases in humans in the future require further exploration. Javanese medaka has proven to be a good model organism that can be utilised in future studies for biomonitoring and elucidating the biomarkers of MPs exposure that may be used as indicator for early human exposure in the future.

## Data Availability

Not applicable.
